# Rare Variants in Antisense Long Noncoding RNA–Protein-Coding Gene Overlap Regions Contribute to Obsessive-Compulsive Disorder

**DOI:** 10.1016/j.bpsgos.2025.100683

**Published:** 2025-12-23

**Authors:** Seulgi Jung, Madison Caballero, Shelby Smout, Behrang Mahjani

**Affiliations:** a Seaver Autism Center for Research and Treatment, Icahn School of Medicine at Mount Sinai, New York, New York; b Department of Psychiatry, Icahn School of Medicine at Mount Sinai, New York, New York; c Department of Genetics and Genomic Sciences, Icahn School of Medicine at Mount Sinai, New York, New York; d Department of Artificial Intelligence and Human Health, Icahn School of Medicine at Mount Sinai, New York, New York; e Mindich Child Health and Development Institute, Icahn School of Medicine at Mount Sinai, New York, New York; f Department of Molecular Medicine and Surgery, Karolinska Institutet, Stockholm, Sweden; g Department of Medical Epidemiology and Biostatistics, Karolinska Institutet, Stockholm, Sweden

**Keywords:** Antisense lncRNAs, Noncoding, OCD Rare variant Whole-genome sequencing

## Abstract

**Background:**

Obsessive-compulsive disorder (OCD) is a prevalent neuropsychiatric disorder with an incompletely understood genetic basis, limiting targeted therapeutic options. Although previous rare-variant studies have primarily focused on protein-coding genes, the contribution of rare regulatory noncoding variants remains largely unexplored.

**Methods:**

We analyzed whole-genome sequencing data from 2561 OCD cases and 12,974 controls from the All of Us Research Program to investigate rare conserved variants within 992 genomic regions where antisense long noncoding RNAs (lncRNAs) overlap protein-coding genes, using both Fisher’s exact test and the Optimal Sequence Kernel Association Test for association testing.

**Results:**

We identified significant enrichment of rare conserved variants in the *KNCN*/*MKNK1-AS1* overlap region in OCD cases (odds ratio = 5.1, false discovery rate < .05). This enrichment was significant in overlapping regions of genes with low evolutionary constraint. Expression analysis revealed strong coexpression of *KNCN* and *MKNK1-AS1* specifically in striatal brain regions (nucleus accumbens: *r* = 0.83, putamen: *r* = 0.81, caudate: *r* = 0.79)—key components of circuits disrupted in OCD. Genes coexpressed with this regulatory pair were enriched for synaptic vesicle dynamics, calcium signaling, and established OCD risk genes from genome-wide association studies (false discovery rate < .05).

**Conclusions:**

These results highlight the importance of rare noncoding regulatory variation in OCD genetics. The association of *KNCN*/*MKNK1-AS1* variants with OCD suggests that antisense lncRNA–protein-coding overlap regions may contribute to disease susceptibility and represent potential therapeutic targets.

## Plain Language Summary

Obsessive-compulsive disorder (OCD) affects millions worldwide, but its genetic basis remains incompletely understood. Previous rare-variant studies focused primarily on deleterious coding variants in protein-coding genes, leaving regulatory elements less explored. We discovered rare conserved non-coding variants in regions where regulatory RNAs overlap with protein-coding genes, particularly affecting *KNCN/MKNK1-AS1*, which shows coordinated activity in brain circuits disrupted in OCD. These variants appear to simultaneously affect both regulatory and protein-coding functions. Our findings reveal a previously unrecognized genetic mechanism contributing to OCD, thereby expanding our understanding of how the disorder develops and identifying potential new directions for research.

Obsessive-compulsive disorder (OCD), characterized by intrusive thoughts and repetitive behaviors, affects 1% to 3% of the global population and typically manifests during childhood. Current therapeutic options offer limited efficacy, partly due to an incomplete understanding of the disorder’s biological basis (1, 2, 3). While cortico-striatal-thalamo-cortical circuits are strongly implicated in OCD pathophysiology (4,5), the genetic mechanisms underlying circuit dysfunction remain poorly understood.

Genome-wide association studies (GWASs) have identified several risk loci, but these explain only a fraction of OCD heritability (6, 7, 8). Rare-variant studies have implicated protein-truncating and -damaging missense variants in constrained genes, as well as rare copy number variants (CNVs) affecting coding regions (9, 10, 11). However, rare-variant approaches have focused primarily on coding sequences, providing limited coverage of noncoding regulatory elements.

Long noncoding RNAs (lncRNAs)—transcripts longer than 200 nucleotides without protein-coding potential—are critical regulators of gene expression through mechanisms including chromatin remodeling, enhancer modulation, and RNA processing (12,13). Approximately 40% of identified lncRNAs display tissue-specific expression in the nervous system, and increasing evidence implicates dysregulated lncRNAs in neuropsychiatric disorders including autism spectrum disorder (14), Rett syndrome (15), schizophrenia (16), and fragile X syndrome (17).

A particularly neglected layer of regulation involves lncRNAs that overlap protein-coding genes in antisense orientation. These transcripts occupy a unique regulatory space: Although noncoding, they overlap exons or introns of protein-coding genes, meaning that a single nucleotide change can affect both transcripts. Antisense lncRNAs modulate sense gene expression through mechanisms including transcriptional interference and epigenetic modifications (18). This dual-transcript architecture makes antisense lncRNAs attractive therapeutic targets, amenable to interventions such as antisense oligonucleotides (19), small interfering RNAs (20), or CRISPR (clustered regularly interspaced short palindromic repeats)–based RNA editing (21), potentially offering enhanced specificity compared with traditional therapies (3,22).

To address this gap, we analyzed whole-genome sequencing (WGS) data from 2561 OCD cases and 12,974 controls in the All of Us Research Program (23). We hypothesized that rare conserved variants within antisense lncRNA–protein-coding gene overlap regions contribute to OCD susceptibility. Furthermore, we hypothesized that identified risk variants would occur in genes showing brain-specific expression patterns relevant to OCD pathophysiology. Our analysis revealed significant enrichment of rare conserved variants in the *KNCN/MKNK1-AS1* overlap region, with functional implications for OCD pathogenesis. These findings illuminate a novel regulatory mechanism in OCD and suggest new avenues for targeted therapeutic development in neuropsychiatric disorders.

## Methods and Materials

### Study Population and Data Acquisition

We analyzed WGS data from the All of Us Research Program (controlled tier, version 8) (23). OCD cases (2764 with WGS data) were identified using ICD-10 codes (F42.0–F42.9), and comorbid psychiatric disorders were assessed (Table S1). For controls, we selected 271,481 individuals without any psychiatric diagnoses (ICD-10 F01–F99) from the WGS dataset.

We performed 1:5 case-control matching based on sex and principal components (PCs) 1 to 5 using the R package optmatch. Genetic ancestry was estimated by the All of Us Research Program using a random forest classifier trained on reference populations; participants were assigned to 6 ancestry groups based on genetic similarity (Figure S1 and Supplemental Methods).

### WGS and Variant Calling

Quality control (QC) procedures began with 2764 cases and 13,820 matched controls. Sample-level filters excluded individuals based on total variant counts, variants absent from gnomAD version 3.1 (24), heterozygous-to-homozygous ratio, call rates, genetic relatedness (kinship coefficient > 0.1 using KING) (25), and sex discrepancies. All of Us used a standardized sequencing platform across genome centers; we excluded all variants carrying any QC flag, including batch-effect flags, to retain only high-quality variants.

Variant-level filters removed low-quality calls based on genotype quality, read depth, allele balance, and quality scores, as well as variants in low-complexity regions, with call rate ≤90% or Hardy-Weinberg equilibrium *p* < 1 × 10^−12^ (detailed filtering criteria are provided in Supplemental Methods). After filtering, 2561 cases and 12,974 controls remained for analysis.

### Annotation of Antisense lncRNAs and Variant Selection

We utilized the Variant Effect Predictor to predict the potential functional effects of the variants (26). To filter rare variants, we included those with an allele count of ≤5 in both our case-control cohort and the nonpsychiatric subset of the gnomAD database version 3.1 (24). Antisense lncRNAs were annotated using the Ensembl database (27). Transcripts were included if they met the following criteria: ≥200 nucleotides in length, lacking protein-coding potential, and transcribed from the antisense strand of annotated protein-coding genes. Rare variants located within antisense lncRNA transcripts and protein-coding genes were kept for analysis.

To enrich for functionally relevant variants, we restricted analysis to evolutionarily conserved positions (GERP++ score > 0) (28), retaining 79,209 rare variants (30% of the initial 263,322 variants) (Supplemental Methods). Sensitivity analyses using stricter thresholds (GERP++ >1, >2, >3) did not substantially alter relative risk (RR) estimates or statistical significance, reinforcing GERP++ > 0 as the optimal threshold (Table S2).

### Statistical Analysis

Rare-variant burden analyses were conducted using Fisher’s exact and binomial tests to compare variant counts between OCD cases and controls. RR ratios and odds ratios (ORs) with 95% CIs were calculated. False discovery rates (FDRs) were controlled using the Benjamini-Hochberg method, with FDR < .05 considered statistically significant.

For gene constraint analysis, genes were stratified into 20 quantiles based on loss-of-function observed/expected upper-bound fraction (LOEUF) scores (24), with lower scores indicating stronger selective constraint. Enrichment analyses were performed within each quantile using binomial tests.

Region-based association testing was performed using the Optimal Sequence Kernel Association Test (SKAT-O) implemented in the SKAT R package (29) (Supplemental Methods). Covariates included sex and PC1 to PC10. Only overlap regions harboring at least 2 rare variants were included. We also used Fisher’s exact test. Due to All of Us data privacy policies, exact carrier counts for rare variants (<20) have not been reported.

### Transcriptomic Analyses

To investigate the correlation between *KNCN* and *MKNK1-AS1* expression across brain tissues, we utilized RNA sequencing data from GTEx version 10 (30). For each of the 13 brain tissues, Pearson correlation coefficients were calculated using log-transformed expression data [log_2_(transcripts per million (TPM) + 1)].

To identify genes coregulated with the *KNCN*/*MKNK1-AS1* pair, we selected 4 brain tissues showing strong coexpression (Pearson’s *r* > 0.7). Within each tissue, genes with median TPM ≥ 0.1 exhibiting strong correlations (|*r*| > 0.7) with both *KNCN* and *MKNK1-AS1* were identified. Gene ontology (GO) enrichment analysis was performed separately for each tissue; enriched biological processes with FDR < .05 were reported (Supplemental Methods).

### Comparison With OCD Risk Genes Identified in GWASs

To evaluate whether OCD-associated genes from the most recent GWAS (251 genes) (6) were enriched among genes showing high correlation with both *KNCN* and *MKNK1-AS1*, we performed Fisher’s exact tests in each of the 4 brain tissues with strong coexpression. For each tissue, we constructed 2 × 2 contingency tables classifying genes by OCD association status and correlation with *KNCN*/*MKNK1-AS1*. Multiple testing correction was performed using the Benjamini-Hochberg procedure (FDR < .05).

As sensitivity analysis, we performed permutation-based enrichment analyses (10,000 permutations per tissue), sampling random gene sets of equal size from the tissue-specific Genotype-Tissue Expression (GTEx) background to obtain empirical null distributions and 1-sided *p* values (Supplemental Methods).

## Results

We analyzed WGS data from 2561 OCD cases and 12,974 ancestry-matched controls from the All of Us Research Program. We identified rare variants (allele count ≤ 5) within genomic intervals where antisense lncRNAs overlapped protein-coding genes, as annotated in GENCODE version 47 (31), excluding protein-truncating and -damaging missense variants affecting the overlapping protein-coding sequences.

To assess potential technical differences between cases and controls across ancestry groups, we compared rare synonymous variant counts (as a proxy for sequencing depth and variant calling sensitivity) and total rare variants with GERP++ scores > 0 within antisense lncRNA–protein-coding gene overlap regions. We performed these analyses in both the complete dataset (*n* = 15,535) and a high-confidence ancestry subset (prediction probability > 0.9, *n* = 10,898). No significant differences were observed between cases and controls in any ancestry group for either metric (binomial test) (Table S3), indicating comparable technical quality and the absence of systematic batch effects between cases and controls within ancestry groups.

Across 992 antisense lncRNA–protein-coding gene overlap regions, we identified 13,091 rare variants in cases and 66,118 rare variants in controls. Initial burden analysis revealed no significant difference in the overall distribution of rare variants between cases and controls (OR = 1.00, binomial *p* = .75).

**Figure 1:**
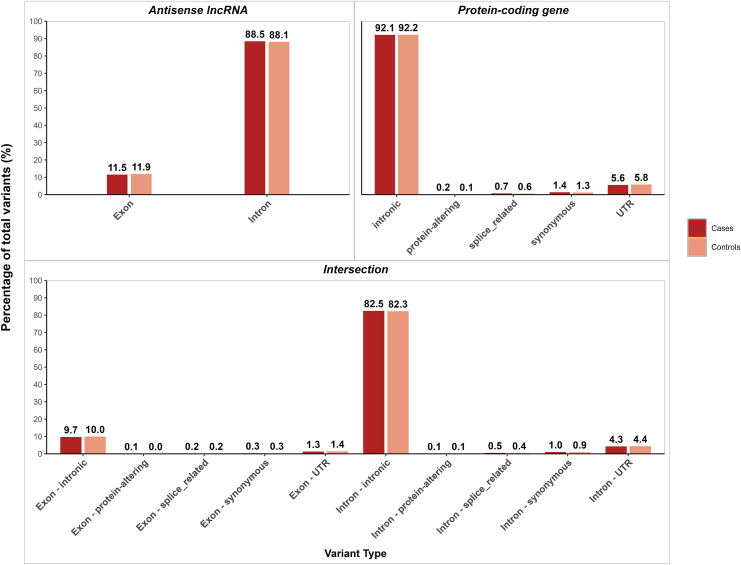
Distribution of rare variant types in antisense lncRNA and protein-coding gene regions in OCD cases and controls. Bar plots showing the percentage of rare variants by functional category in antisense lncRNA regions, protein-coding gene regions, and overlapping regions between antisense lncRNAs and protein-coding genes (intersection). Dark red bars represent OCD cases; light red bars represent controls. Variant categories include exonic variants, intronic variants, and splice-related variants. Numbers above bars indicate the percentage of total variants in each category. No significant differences were observed between cases and controls for any variant type across all 3 genomic contexts (binomial *p* > .05). The analysis included 79,209 rare variants that passed quality control filters and showed evidence of evolutionary conservation (GERP++ > 0) across 992 antisense lncRNA–protein-coding gene overlap regions. lncRNA, long noncoding RNA; OCD, obsessive-compulsive disorder; UTR, untranslated region.

We examined the distribution of rare variant types across antisense lncRNA regions, protein-coding gene regions, and their overlapping intervals to identify potential enrichment patterns in OCD cases versus controls (Figure 1 and Table S4). The variant spectrum was dominated by intronic variants in all 3 genomic contexts, comprising 88% of variants in antisense lncRNAs, 92% in protein-coding genes, and 82% of intronic variants in both antisense lncRNAs and protein-coding genes. We observed no significant differences in the distribution of any variant type between cases and controls across all 3 genomic contexts (binomial *p* > .05).

To investigate whether the constraint level of protein-coding genes influences the burden of rare variants in antisense lncRNA overlap regions, we stratified genes into 20 quantiles based on their LOEUF scores (24). LOEUF quantifies gene-level constraint, with lower scores indicating greater intolerance to loss-of-function variation, suggesting constrained genes. We checked per-window gene and variant counts in Table S5. Coverage varied substantially across LOEUF windows: The number of genes per window ranged from 10 to 103, and the corresponding number of rare conserved variants ranged from 372 to 9610. Thus, statistical power is not uniform across LOEUF bins and should be taken into account when interpreting the observed enrichment patterns.

**Figure 2:**
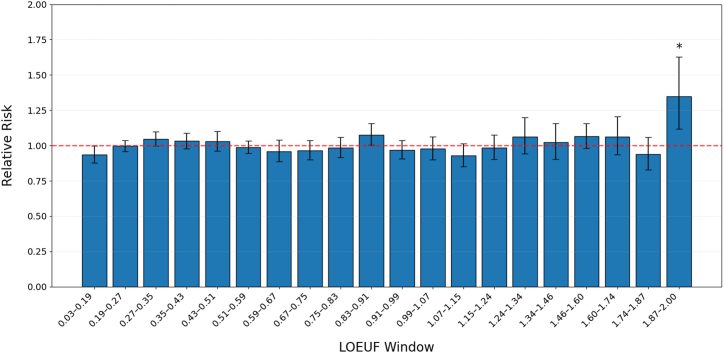
Enrichment of rare conserved variants within the overlapping region of antisense long noncoding RNA and their sense protein-coding genes in OCD. Relative risk of rare conserved variants in the overlapping region across 20 equally spaced quantiles of LOEUF scores. The first quantile includes most constrained genes. The figure shows risk ratios with 95% CIs. The dashed horizontal line represents relative risk = 1, indicating no effect. Bars above this line suggest increased risk. ∗*p* < .05 using a binomial test between OCD cases and unaffected controls. LOEUF, loss-of-function observed/expected upper bound fraction; OCD, obsessive-compulsive disorder.

Notably, we observed a significant enrichment of rare conserved variants in OCD cases, specifically within overlap regions of the least constrained genes (highest LOEUF quantile: RR = 1.35; 95% CI, 1.12–1.63; binomial *p* = .003) (Figure 2). This pattern remained consistent in a sensitivity analysis restricted to individuals of European ancestry involving 1947 cases and 9937 controls (RR = 1.43; 95% CI, 1.13–1.81; binomial *p* = .004) (Figure S2).

To assess whether the pattern across LOEUF bins could be an artifact of differential statistical power, we implemented a fixed-K bootstrap with replacement within each bin (K = 10). Despite wider bootstrap intervals in sparsely represented bins, the central tendency of RR remained consistent with the main result, indicating that the cross-bin pattern is not driven solely by differences in statistical power (Figure S3A, B).

To examine whether the enrichment of rare variants in the least constrained genes was influenced by evolutionary conservation stringency (GERP++ score), we repeated our analysis using increasingly strict conservation thresholds (Figure S4A–E). As we applied more stringent conservation filters, we observed a progressive increase in RR for variants in the 20th LOEUF quantile.

### Antisense lncRNA and Protein-Coding Gene Pairs Associated With the Risk of OCD

To identify specific antisense lncRNA–protein-coding gene overlap regions associated with OCD risk, we performed comprehensive rare-variant association analyses using both Fisher’s exact and SKAT-O tests on 2561 OCD cases and 13,517 controls to ensure robust detection across different genetic architectures (29). We analyzed cumulative rare-variant burden across 992 overlapping regions between antisense lncRNAs and their corresponding protein-coding genes (Table S6).

**Figure 3:**
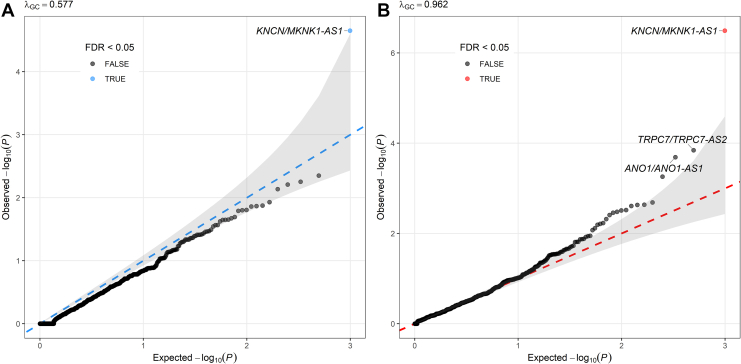
QQ plots of rare variant association tests in antisense long noncoding RNA–protein-coding gene overlap regions. The QQ plot displays the observed −log_10_(*p*) values against the expected −log_10_(*p*) values under the null hypothesis. The QQ plots show the distribution of association *p* values for rare variant burden analysis in antisense lncRNA–protein-coding gene overlap regions. **(A)** Fisher’s exact test results across 992 overlap regions (λ_GC_ = 0.577). **(B)** Optimal Sequence Kernel Association Test results for the same regions (λ_GC_ = 0.962). In both panels, the diagonal dashed line represents the null expectation of no association, with the gray shaded area indicating the 95% CI. Points are colored based on FDR significance: Blue/red points indicate FDR < .05, while gray points indicate FDR ≥ .05. The names of gene pairs with FDR < .1 are labeled. FDR, false discovery rate; QQ, quantile-quantile.

The quantile-quantile plots revealed deflated genomic inflation factors in both analyses (Fisher’s exact test: λ_GC_ = 0.577; SKAT-O: λ_GC_ = 0.962), with values below the expected null of 1.0 (Figure 3A, B). This deflation likely reflects the sparsity of rare conserved variants within individual overlap regions and the focused nature of our analysis on specific genomic features (antisense lncRNA overlaps), where only a subset may be functionally relevant to OCD. The SKAT-O test showed less deflation than Fisher’s exact test, suggesting that it may be more powerful for detecting associations when analyzing sparse variant data. Among all tested regions, a gene pair (*KNCN/MKNK1-AS1*) showed a significant association after multiple testing corrections using FDR < .05 (Table 1 and Figure 3A, B).

**Table 1:** Top Five Overlapping Regions in Fisher’s Exact Test and SKAT-O Between 2561 Obsessive-Compulsive Disorder Cases and 12,974 Controls

Overlapping Region		Protein-Coding Gene		Antisense lncRNA		Fisher’s Exact Test			SKAT-O Test	
Chr	Position	Symbol	Position	Symbol	Position	OR	*p*	*p*_FDR_	*p*	*p*_FDR_
1	46,545,641–46,551,647	*KNCN*	46,545,641–46,551,647	*MKNK1-AS1*	46,538,611–46,570,255	5.1	2.24 × 10^−5^	.02	3.22 × 10^−7^	.0003
5	136,303,757–136,316,100	*TRPC7*	136,212,745–136,365,545	*TRPC7-AS2*	136,303,757–136,316,100	1.7	5.60 × 10^−3^	1.00	1.43 × 10^−4^	.0677
11	70,187,788–70,188,509	*ANO1*	69,985,907–70,189,530	*ANO1-AS1*	70,187,788–70,188,509	Inf	4.48 × 10^−3^	1.00	2.05 × 10^−4^	.0677
3	85,992,183–86,028,007	*CADM2*	84,958,989–86,074,429	*CADM2-AS1*	85,992,183–86,028,007	1.4	1.36 × 10^−2^	1.00	5.52 × 10^−4^	.1369
7	79,008,988–79,012,277	*MAGI2*	78,017,055–79,453,667	*MAGI2-AS2*	79,008,988–79,012,277	3.6	1.38 × 10^−2^	1.00	2.06 × 10^−3^	.2880

Inf denotes an infinite OR, indicating that rare variants were observed exclusively in cases, and none were found in controls. FDR correction was performed using the Benjamini-Hochberg procedure. Position indicates genomic position (hg38).

Chr, chromosome; FDR, false discovery rate; Inf, infinity; lncRNA, long noncoding RNA; OR, odds ratio; SKAT-O, Optimal Sequence Kernel Association Test.

No other overlapping region achieved an FDR < .05 in either test. Given that the overall rare-variant burden across all 992 overlaps was not elevated in cases (OR = 1.00, binomial *p* = .75), this result reinforces that the risk is localized to specific loci.

As a sensitivity analysis, we repeated the same analyses restricted to individuals of European ancestry (1947 cases and 9937 controls). The *KNCN*/*MKNK1-AS1* gene pair remained significant at FDR < .05 in both Fisher’s exact test (FDR = .02) and the SKAT-O test (FDR = .004) (Table S7).

*KNCN* encodes kinocilin, highly expressed in brain tissues, while *MKNK1-AS1* is an antisense lncRNA that overlaps with both *KNCN* and *MKNK1* (Figure S5). *MKNK1-AS1* is located on the forward (plus) strand (chromosome 1:46,538,611–46,570,256, GRCh38); however, both *KNCN* (chromosome 1:46,545,641–46,551,647) and *MKNK1* (chromosome 1:46,557,407–46,616,843) are located on the reverse (minus) strand. Specifically, the *MKNK1-AS1* transcript completely encompasses the genomic locus of *KNCN*. As a result, variants identified in this region reside simultaneously within the *MKNK1-AS1* transcript and the *KNCN* gene body, creating a configuration where a single nucleotide change may influence the regulation or transcription of both sense and antisense RNA molecules. Rare variants associated with OCD were located at the 5′ end, the 3′ end, and intronic sequences of *KNCN*, as well as intronic regions of *MKNK1-AS1* (Figure S5).

Notably, rare conserved variants in the *KNCN/MKNK1-AS1* overlap region are within candidate cisregulatory elements identified by ENCODE as having a high likelihood of being involved in regulating gene expression (32), transcription binding sites identified by JASPAR (33), and regulatory region identified by the open regulatory annotation database (Figure S5) (34). Single nucleotide polymorphisms in this overlapping region showed expression quantitative trait loci (eQTL) effects on *KNCN*, *MKNK1-AS1*, *MKNK1*, *MOB3C*, *TMEM275*, *FOXD2*, and *EFCAB14* in brain and whole-blood tissues (Table S8).

Two additional overlap regions showed suggestive evidence of an association (FDR < .1 in SKAT-O test) and warrant further investigation as candidate risk loci (Table 1 and Figure 3B). The *TRPC7/TRPC7-AS2* region (FDR = .0677 in SKAT-O) contains *TRPC7*, which encodes a calcium-permeable cation channel crucial for neuronal excitability and implicated in seizure susceptibility and circadian rhythm regulation (35). The *ANO1/ANO1-AS1* region (FDR = .0677 in SKAT-O) harbors *ANO1*, encoding a calcium-activated chloride channel essential for neuronal signaling (36). The near-significant associations of these functionally relevant genes suggest that they may contribute to OCD pathophysiology through disrupted ion channel function and neuronal excitability.

### Gene Expression Correlation Analysis

**Figure 4:**
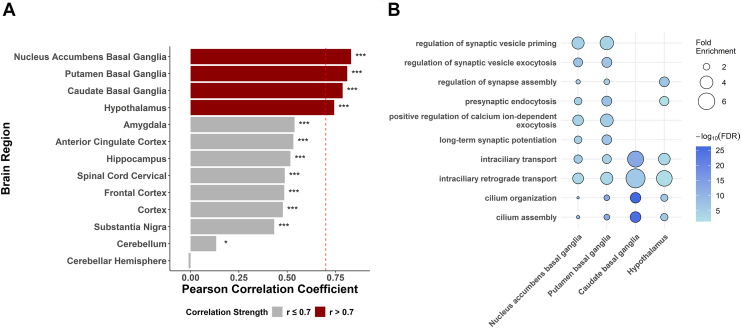
Brain region–specific coexpression analysis of *KNCN* and *MKNK1-AS1* and functional pathway enrichment. **(A)** Pearson correlation coefficients between *KNCN* and *MKNK1-AS1* expression levels across 13 brain regions from the GTEx version 10 dataset. Brain regions are ordered by correlation strength. Dark red bars indicate strong positive correlations (*r* > 0.7), while gray bars indicate moderate to weak correlations (*r* ≤ 0.7). The dashed red line marks the *r* = 0.7 threshold. Significance levels are denoted as ∗*p* < .05, ∗∗∗*p* < .001. **(B)** Gene ontology enrichment analysis of biological processes for genes showing strong positive correlation (*r* > 0.7) with both *KNCN* and *MKNK1-AS1* in the 4 brain regions with highest coexpression (nucleus accumbens, putamen, caudate, and hypothalamus). Circle size represents fold enrichment, and color intensity indicates −log_10_(FDR) values, with darker blue representing more significant enrichment. FDR, false discovery rate.

To investigate the potential regulatory relationship between the antisense lncRNA *MKNK1-AS1* and its overlapping protein-coding gene *KNCN*, we performed correlation analysis of their expression patterns across 13 brain regions from the GTEx database version 10 (Figure 4A and Figure S6A–M). We observed substantial variation in the strength of coexpression between these genes across different brain tissues.

A total of 12 brain regions showed positive correlation with *p* < .05 (Figure 4A and Figure S6A–M). The strongest positive correlations (*r* > 0.7) were observed in 4 brain regions critical for OCD pathophysiology: the nucleus accumbens (*r* = 0.83), putamen (*r* = 0.81), caudate (*r* = 0.79), and hypothalamus (*r* = 0.74). Notably, all 3 components of the striatum (the nucleus accumbens, putamen, and caudate) demonstrated remarkably high coexpression between *KNCN* and *MKNK1-AS1*, suggesting coordinated regulation within the basal ganglia circuitry.

To assess whether this strong striatal coexpression was atypical relative to other antisense pairs, next we computed Pearson correlation coefficients for all 992 antisense lncRNA–protein-coding gene pairs in each GTEx striatal tissue. The pair of *KNCN*/*MKNK1-AS1* ranked in the 90.7th, 94.1st, and 90.5th percentiles of the correlation distributions in the caudate, nucleus accumbens, and putamen, respectively (Figure S7A–C), indicating that its striatal coexpression is stronger than that observed for the vast majority of antisense lncRNA–protein-coding gene pairs. These benchmarking analyses support that the *KNCN*/*MKNK1-AS1* transcript pair shows relatively strong and specific coregulation in striatal circuits rather than reflecting a generic feature of antisense lncRNA–protein-coding gene organization.

To identify biological processes potentially influenced by the *KNCN/MKNK1-AS1* regulatory axis, we performed GO enrichment analysis using genes that showed strong positive correlation (*r* > 0.7) with both *KNCN* and *MKNK1-AS1* expression in the 4 brain regions with the highest coexpression (Table S9). This analysis revealed significant enrichment for synaptic and neuronal function pathways, particularly those related to synaptic vesicle dynamics (regulation of synaptic vesicle priming, regulation of synaptic vesicle exocytosis), synaptic plasticity (long-term synaptic potentiation), and calcium signaling (positive regulation of calcium ion–dependent exocytosis) (Figure 4B and Table S10). Additionally, we observed enrichment for cilium-related processes (cilium organization and cilium assembly) and intracellular transport mechanisms (intraciliary transport and intraciliary retrograde transport), suggesting potential roles in primary cilia function and cellular trafficking.

### Enrichment Analysis of OCD Risk Genes

In the most recent OCD GWAS by Strom *et al.* (6), neither *KNCN* nor *MKNK1* was among the 251 prioritized risk genes, and neither gene showed a genome-wide significant association at the single-variant or gene-based level. To determine whether OCD risk genes were enriched among genes coexpressed with *KNCN* and *MKNK1-AS1*, we used the 251 genes identified in the current OCD GWAS (6) as OCD risk genes and performed Fisher’s exact tests comparing their proportion within high-correlation gene sets versus tissue-specific background genes. All 4 brain regions showed significant enrichment of OCD risk genes with FDR < .05 among genes highly correlated with both *KNCN* and *MKNK1-AS1* expressions (Table 2). The nucleus accumbens demonstrated the most significant enrichment, followed by the caudate, hypothalamus, and putamen. The OCD risk genes overlapping with *KNCN*/*MKNK1-AS1* coexpressed genes in the 4 brain tissues are described in Table S11.

**Table 2:** Enrichment of Obsessive-Compulsive Disorder Risk Genes Among Genes Highly Correlated With Both *KNCN* and *MKNK1-AS1* Expression

Tissue	Pearson’s *r* > 0.7		Pearson’s *r* < 0.7		Fisher’s Exact Test			Permutation Test	
	Risk Genes	Nonrisk Genes	Risk Genes	Nonrisk Genes	*p*	OR	*p*_FDR_	*p*	*p*_FDR_
Nucleus Accumbens Basal Ganglia	74	6269	107	18,658	2.79 × 10^−6^	2.06	1.12 × 10^−5^	1.00 × 10^−4^	4.00 × 10^−4^
Caudate Basal Ganglia	32	2333	148	22,148	4.58 × 10^−4^	2.05	9.16 × 10^−4^	5.00 × 10^−4^	1.00 × 10^−3^
Putamen Basal Ganglia	56	5029	122	18,473	1.18 × 10^−3^	1.69	1.58 × 10^−3^	1.20 × 10^−3^	1.60 × 10^−3^
Hypothalamus	26	1991	158	23,097	3.15 × 10^−3^	1.91	3.15 × 10^−3^	3.60 × 10^−3^	3.60 × 10^−3^

FDR, false discovery rate; OR, odds ratio.

To validate these results, we performed permutation-based enrichment analyses (10,000 permutations per tissue). Empirical *p* values closely matched Fisher’s exact test *p* values and remained significant across all 4 brain regions (Table 2).

## Discussion

Our findings extend prior rare-variant work in OCD by implicating a distinct class of genetic risk. Earlier studies emphasized rare protein-truncating and -damaging missense variants in constrained genes, as well as rare CNVs disrupting coding regions (9, 10, 11). By design, those approaches capture the consequences of severe coding disruption but only partially survey noncoding regulatory architectures. In contrast, our analysis of conserved variants in antisense lncRNA–protein-coding overlap regions identifies a regulatory mechanism where a single variant can perturb both transcripts. This architecture is not well covered by standard exome or CNV pipelines, indicating that these signals represent an orthogonal layer of genetic risk rather than replication of known coding associations. This study provides systematic evidence linking rare conserved variants in antisense lncRNA–protein-coding gene overlap regions to OCD risk. Our findings reveal a genetic architecture where single nucleotide variants can simultaneously affect both regulatory lncRNAs and their overlapping protein-coding genes, potentially amplifying their functional impact on disease susceptibility.

The identification of *KNCN/MKNK1-AS1* as the top association signal is particularly intriguing, given the biological functions of these genes. *KNCN* encodes kinocilin, a protein involved in stabilizing dense microtubular networks or in vesicular trafficking (37), while *MKNK1* encodes a kinase that regulates translation in response to synaptic activity (38). The antisense lncRNA *MKNK1-AS1* overlaps both genes, creating a complex regulatory unit where variants could disrupt multiple aspects of neuronal function simultaneously. The localization of OCD-associated variants to conserved regulatory elements within this overlap region, coupled with their eQTL effects on multiple genes, suggests that these variants may alter the coordinated expression of genes critical for neural circuit function.

Notably, the *KNCN*/*MKNK1-AS1* locus was not identified in previous exome or CNV analyses (9, 10, 11), supporting that it reflects a noncoding regulatory mechanism uniquely detectable in WGS data. Differences in study design mean that direct locus-by-locus replication of earlier hits is not expected; rather, our results add a mechanistically distinct, antisense lncRNA-mediated component to the emerging picture of rare genetic risk in OCD, complementing rather than contradicting the existing coding-focused literature.

The gene constraint patterns observed here also complement prior findings. Rare coding variant studies in OCD and neuropsychiatric disorders have identified enrichment in genes with low LOEUF scores (9,10,39, 40, 41, 42, 43), whereas our analyses reveal enrichment in antisense-overlap regions of the least constrained genes (highest LOEUF). One interpretation is that highly constrained genes are primarily vulnerable to coding loss-of-function variation, whereas less constrained genes may depend more on precise regulatory control of transcript dosage. Thus, coding variants in constrained genes and regulatory antisense-overlap variants in less constrained genes may provide complementary routes to OCD risk.

The brain region–specific expression analysis revealed remarkably high coexpression between *KNCN* and *MKNK1-AS1* in striatal regions (nucleus accumbens, putamen, and caudate), which are key components of the cortico-striato-thalamo-cortical circuits implicated in OCD, and in the hypothalamus, a functionally connected region involved in stress and arousal regulation. This spatial specificity suggests that the *KNCN/MKNK1-AS1* regulatory axis may be particularly important for the development and function of circuits disrupted in OCD. A recent functional imaging study demonstrated that altered resting-state connectivity specifically involving the putamen, a critical striatal region highlighted by our coexpression analyses, was associated with obsessive-compulsive symptoms in adolescents, particularly under conditions of increased environmental stress (44). These converging genetic and neuroimaging results suggest that dysregulated molecular and functional interactions in the striatum may contribute to OCD pathophysiology. Additionally, the enrichment of OCD risk genes among genes coexpressed with this regulatory pair further strengthens the connection to disease pathophysiology.

The functional pathways enriched among *KNCN/MKNK1-AS1* highly coexpressed genes, including synaptic vesicle dynamics, calcium signaling, and cilium organization, align well with emerging models of OCD pathogenesis. Disrupted synaptic transmission and calcium homeostasis have been implicated in OCD through both human genetics and animal models (45, 46, 47). The unexpected enrichment of cilium-related processes is particularly intriguing, as primary cilia play crucial roles in neuronal development and signaling, although their involvement in OCD has not been explored previously (48).

Several limitations of the current study should be acknowledged. First, while our sample size was sufficient to detect the strongest associations, the deflated genomic inflation factors suggest that we may be underpowered to detect additional loci with smaller effect sizes. Larger cohorts will be needed to comprehensively map the contribution of antisense lncRNA variants to OCD risk. Second, our analysis focused on rare variants with evolutionary conservation, potentially missing functionally important variants in less conserved regions. Third, the functional consequences of the identified variants require experimental validation through cellular and animal models. Fourth, our use of psychiatrically healthy control individuals might have introduced bias if rare variants in antisense overlapped with regions that confer transdiagnostic risk across multiple psychiatric disorders. Carriers of these variants who develop other psychiatric conditions would be underrepresented in control individuals, potentially inflating effect sizes. Therefore, our results should be interpreted as evidence for enrichment in OCD cases relative to psychiatrically healthy controls rather than proof of OCD specificity. Disentangling disorder-specific versus transdiagnostic effects will require future studies that include psychiatric comparison groups, allowing direct tests of whether the observed enrichment at loci such as *KNCN*/*MKNK1-AS1* is unique to OCD or reflects a broader neuropsychiatric vulnerability. Fifth, the observed expression correlations between antisense lncRNA and protein-coding gene pairs, as identified from GTEx bulk tissue data, highlight potential regulatory relationships; however, we acknowledge that these correlations alone do not definitively establish causative regulatory mechanisms. It remains possible that the strong coexpression signals represent either direct cisregulatory interactions mediated by antisense transcription or indirect coregulation through shared transcription factors, enhancer elements, or epigenetic marks. Future functional validation experiments, such as targeted genome editing or antisense RNA knockdown studies, will be critical to confirm direct regulatory causation and delineate the precise molecular mechanisms underpinning these observed expression correlations.

Future studies should prioritize functional characterization of the *KNCN/MKNK1-AS1* variants using patient-derived induced pluripotent stem cells and brain organoids. CRISPR-based editing could be used to model specific variants and assess their effects on gene expression, neuronal development, and circuit function. Additionally, investigating whether antisense lncRNA variants influence OCD symptom dimensions, treatment response, or disease trajectory could enable more personalized therapeutic approaches.

### Conclusions

By focusing on antisense lncRNA overlap regions, we identified a novel OCD risk locus. The *KNCN/MKNK1-AS1* association, combined with its striatal-specific expression patterns and coexpression with OCD risk genes, provides new insights into the molecular mechanisms underlying OCD. These findings expand our understanding of the genetic architecture of neuropsychiatric disorders beyond protein-coding variation and highlight antisense lncRNAs as promising targets for next-generation therapeutics. As we move toward precision medicine approaches for psychiatric disorders, targeting specific regulatory RNAs based on individual genetic profiles may offer more effective and personalized treatment strategies for OCD and related conditions.

## Supplementary Material

Tables S1–S11

Key Resources Table
