# Supplementary material for: Rare Variants in Antisense Long Noncoding RNA–Protein-Coding Gene Overlap Regions Contribute to Obsessive-Compulsive Disorder
Source: Biol Psychiatry Glob Open Sci. 2025 Dec 23;6(2):100683. doi: 10.1016/j.bpsgos.2025.100683 (PMC12887771; doi:10.1016/j.bpsgos.2025.100683)
Supplement: Supplemental Methods and Figures S1–S7 [file mmc1.pdf]

SUPPLEMENTARY INFORMATION

Rare Variants in Antisense Long Non-Coding RNA–Protein-Coding Gene Overlap Regions Contribute to Obsessive-Compulsive Disorder

Jung *et al.*

Supplementary Methods

Study population and data acquisition

The All of Us Research Program, led by the National Institutes of Health (NIH), is a pioneering precision medicine initiative designed to advance health research and medical discoveries by collecting diverse data from at least one million participants across the United States(1). In the current All of Us Research Program workspace (controlled tier, version 8), we identified samples diagnosed with OCD, of which 2,764 had available whole-genome sequencing (WGS) data. We assessed the prevalence of comorbid psychiatric disorders using ICD-10 diagnostic codes (Supplementary Table 1). OCD diagnoses were identified using International Classification of Diseases, 10th Revision (ICD-10) codes (F42.0–F42.9). For case-control matching, we selected these 2,764 OCD cases and 271,481 individuals without mental disorders (ICD-10 F01-F99) from the WGS dataset. To reduce phenotypic heterogeneity in the control group, we excluded individuals with any recorded psychiatric diagnoses from the pool of potential controls. This "psychiatrically healthy" control definition was chosen to minimize the likelihood of including individuals with undiagnosed or subsyndromal OCD, which could dilute case–control differences and reduce power.

Using the R package optmatch (https://CRAN.R-project.org/package=optmatch), we performed 1:5 case-control matching based on sex and principal components (PCs) 1–5, which were derived from WGS data and provided by the All of Us Research Program.

Genetic ancestry was estimated by the All of Us Research Program using a random forest classifier trained on reference populations from the Human Genome Diversity Project (HGDP) and 1000 Genomes Project (N=3,942). The classifier used the first 16 principal components derived from high-quality autosomal variants as features. Participants were assigned to ancestry groups based on genetic similarity to reference populations: African (AFR), Americas (AMR), East Asian (EAS), European (EUR), Middle Eastern (MID), and South Asian (SAS). Each sample was assigned to the ancestry with the highest prediction probability (Supplementary Figure 1A and B).

Whole-genome sequencing and variant calling

We started quality control (QC) procedures using 2,764 cases and 13,820 matched controls. Individuals were excluded if they had fewer than 2.4 million or more than 5.0 million total variants, over 100,000 variants absent from gnomAD v3.1(2), or a heterozygous-to-homozygous variant ratio exceeding 3.3. All of Us used a fully standardized platform across the three genome centers, including the same NovaSeq 6000 instrument, PCR-free Kapa HyperPrep library prep, DRAGEN v3.7.8 software, and identical configurations. Even with this uniform setup, the program evaluated batch effects arising from center-specific differences, the 192 structural-variant calling batches, and sample type (blood vs saliva). Structural-variant batches showing significant effects were marked with the “VARIABLE_ACROSS_BATCHES” filter. In our analysis, we excluded all variants carrying any QC flag, including batch-effect flags, to retain only high-quality variants.

We further filtered out low-quality variants based on the following criteria: genotype quality (GQ) ≤ 20, read depth (DP) ≤ 10, allele balance (AB) ≤ 0.2 for heterozygotes, ExcessHet < 54.69, and quality scores (QUAL) < 60 for single nucleotide variants (SNVs) and < 69 for short insertions and deletions (InDels). Variants located in low-complexity regions were also removed. Additional QC steps excluded samples with call rates more than three standard deviations below the mean, individuals with close genetic relatedness (kinship coefficient > 0.1) based on Kinship-based INference for Genome-wide association studies (KING)(3), and those with sex discrepancies.

After filtering, 2,561 cases and 12,974 controls remained. Further exclusions included homozygous reference calls with < 90% read depth supporting the reference allele or GQ < 25, heterozygous calls with AB < 30%, GQ < 25, or a binomial probability > 1×10^{-9} for the observed allele balance centered at 0.5, and homozygous alternate calls with < 90% read depth supporting the alternate allele or GQ < 25. Variants with a call rate ≤ 90% and Hardy-Weinberg equilibrium $P < 1\times10^{-12}$ were also removed.

To enrich for functionally relevant variants, we utilized the genomic evolutionary rate profiling (GERP++) score, which quantifies evolutionary constraint at genomic positions based on conservation across species, with higher scores indicating stronger conservation and potential functional importance(4).

We restricted our analysis to evolutionary conserved positions, defined as having a GERP++ score > 0. This conservation filter retained 79,209 rare variants (30% of the initial 263,322 variants), effectively balancing sensitivity with specificity to maintain adequate statistical power. To evaluate how different conservation stringency thresholds

might influence our findings, we conducted sensitivity analyses using stricter thresholds (GERP++ > 1, > 2, and > 3). Higher conservation thresholds significantly reduced variant numbers, retaining fewer than 10% of variants at GERP++ > 1 (Supplementary Table 2). These stricter thresholds did not substantially alter the overall relative risk estimates or the statistical significance of our primary findings, reinforcing our selection of GERP++ > 0 as the optimal threshold.

Statistical analysis

Rare variant burden analyses were conducted using Fisher's exact tests to compare the cumulative number of rare variants in each antisense lncRNA between OCD cases and controls. Relative risk ratios (RRs) with 95% confidence intervals (CIs) were calculated to quantify the enrichment of rare variants in OCD cases relative to controls using binomial tests. False discovery rates (FDRs) were controlled using the Benjamini–Hochberg method, with an FDR threshold of < 0.05 defining statistically significant associations. Odds ratios (ORs) and 95% CIs were also computed to further quantify associations.

For gene constraint analysis, cognate sense genes were stratified into 20 equally sized quantiles based on their loss-of-function observed/expected upper bound fraction (LOEUF) scores (range: 0–2, where lower scores indicate stronger selective constraint)(2). Enrichment analyses were performed within each decile to identify differential burden patterns among OCD-associated antisense lncRNAs using binomial tests.

The antisense lncRNA-protein coding gene overlap region -based association testing was performed using the optimal unified sequence kernel association test (SKAT-O) implemented in the SKAT R package (5). SKAT-O combines the burden test and SKAT through an optimal linear combination, providing robust power across different genetic architectures. The test adaptively selects the optimal combination parameter that maximizes statistical power with the covariate matrix including sex and PC1-PC10. Only the overlapping regions harboring at least two rare variants (allele count ≤ 5) were included in the analysis. Three test statistics were computed for each gene: (1) SKAT *P* value, optimal for scenarios where variants have different directions of effect; (2) Burden test *P* value, optimal when variants have the same direction of effect; and (3) SKAT-O *P* value, which adaptively combines both tests.

To account for multiple testing across the overlapping regions, we applied two correction methods. The Bonferroni correction was calculated as α/n, where $\alpha = 0.05$ and n represents the number of the overlapping regions tested. Additionally, the false discovery rate (FDR) was controlled using the Benjamini-Hochberg procedure. Genes with FDR <0.05 were considered statistically significant.

We also employed Fisher's exact test for the overlapping region-based rare variant association testing. Fisher's exact test compares the proportion of individuals carrying at least one rare variant within each overlap region between cases and controls, assuming all variants contribute similarly to disease risk. Due to All of Us Research Program data privacy policies regarding small cell counts (<20), exact carrier counts for rare variants cannot be displayed.

Transcriptomic analyses

To investigate the correlation between *KNCN* and *MKNK1-AS1* gene expression across multiple brain tissues, RNA-seq data from the Genotype-Tissue Expression (GTEx) v10 dataset were utilized(6). For each of the 13 brain tissues, gene expression data were obtained in transcripts per million (TPM) format. Pearson correlation coefficients were calculated for each tissue to quantify the relationship between *KNCN* and *MKNK1-AS1* expression using "cor.test" function in R. For the correlation analysis, expression data for each tissue were log-transformed (log2(TPM + 1)) to normalize distribution. Statistical significance of the correlations was determined by performing Pearson's correlation tests.

To identify genes demonstrating strong correlation with both *KNCN* and *MKNK1-AS1* expression across four brain tissues (the nucleus accumbens, hypothalamus, putamen, and caudate) with Pearson $r > 0.7$ between *KNCN* and *MKNK1-AS1* expression, we conducted a comprehensive correlation analysis. We calculated Pearson correlation coefficients between the expression of each gene and both *KNCN* and *MKNK1-AS1* individually within each tissue. Genes, with median TPM ≥ 0.1, exhibiting strong correlations (|Pearson r| > 0.7) with both *KNCN* and *MKNK1-AS1* were identified and saved separately per tissue.

We then performed Gene Ontology (GO) enrichment analysis separately for each tissue using its respective set of high-correlation genes. This tissue-specific approach allowed us to identify biological processes that may be regulated by the *KNCN/MKNK1-AS1* axis in a region-dependent manner. Enriched GO terms were filtered for biological processes

with FDR < 0.05 and compared across tissues to identify both shared and tissue-specific pathways.

Comparison with OCD risk genes identified in GWAS

The current GWAS identified 251 genes associated with OCD risk(7). To evaluate whether OCD-associated genes were significantly enriched among genes showing high correlation with both *KNCN* and *MKNK1-AS1* expression in the four brain tissues (the nucleus accumbens, hypothalamus, putamen, and caudate), we performed tissue-specific enrichment analyses using Fisher's exact test. For each tissue, we first identified the set of genes, with median TPM ≥ 0.1, demonstrating strong correlation (|Pearson r| > 0.7) with both *KNCN* and *MKNK1-AS1* expressions as described above.

We constructed a 2 × 2 contingency table for each tissue, classifying genes according to two criteria: (1) whether they were OCD-associated genes and (2) whether they showed high correlation with both *KNCN* and *MKNK1-AS1*. The contingency table comprised four categories: (a) OCD-associated genes present in the high-correlation set; (b) non-OCD genes present in the high-correlation set; (c) OCD-associated genes not present in the high-correlation set but expressed in the tissue; and (d) non-OCD genes expressed in the tissue but not in the high-correlation set.

Using Fisher’s exact test, we assessed whether the overlap between the OCD gene set and the high-correlation gene set for each tissue was greater than expected by chance. We corrected for multiple testing across the four tissues using the Benjamini-Hochberg

procedure to control the FDR. Enrichment results with an FDR-adjusted *P* value < 0.05 were considered statistically significant.

For the sensitivity analysis, we additionally performed permutation-based enrichment analyses. For each tissue, we preserved the size of the high-correlation gene set and repeatedly sampled random gene sets of the same size from the tissue-specific GTEx background (10,000 permutations), computing the number of overlapping OCD risk genes in each permuted set to obtain an empirical null distribution of overlaps. Empirical one-sided *P* values, calculated as b/10,000 where b is the number of permutations with overlap greater than or equal to the observed value.

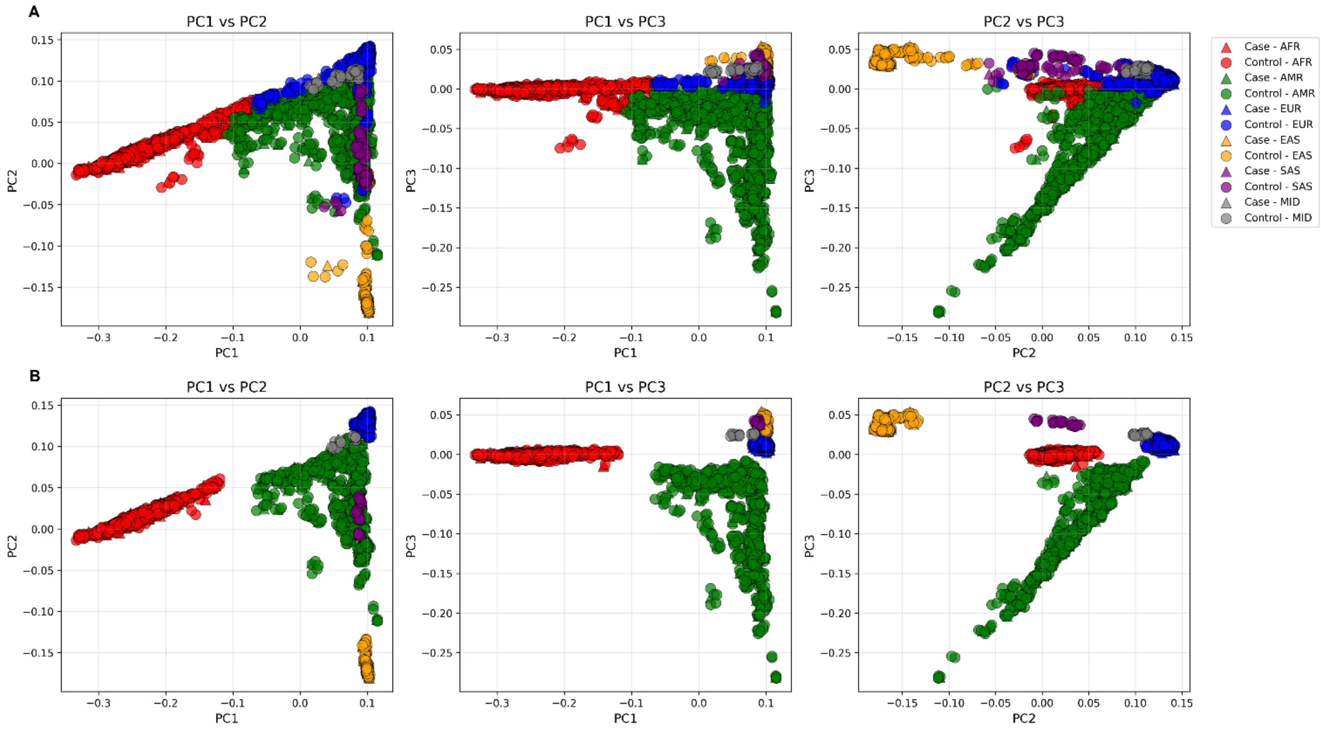

Supplementary Figure 1. Principal component analysis of OCD cases and controls across genetic ancestry groups. Genetic ancestry was inferred by measuring genetic similarity of each participant to global reference populations using a random forest classifier trained on harmonized continental metadata labels from the Human Genome Diversity Project (HGDP) and 1000 Genomes Project (N=3,942 samples). **(A)** All samples (n=15,535; cases=2,561, controls=12,974), with each participant assigned to the genetic ancestry group with the highest classifier probability. Cases are represented by triangles and controls by circles. Colors indicate genetic ancestry groups: AFR (African, red; 285 cases, 1,375 controls), AMR (Admixed-American, green; 270 cases, 1,371 controls), EAS (East Asian, orange; 33 cases, 165 controls), EUR (European, blue; 1,947 cases, 9,937 controls), MID (Middle Eastern, grey; 12 cases, 54 controls), and SAS (South Asian, purple; 14 cases, 72 controls). **(B)** High-confidence ancestry assignments (n=10,898; cases=2,123, controls=8,775), filtered to include only samples where the maximum classifier probability exceeded 0.9, demonstrating reduced admixture and tighter population structure clustering. AFR (247 cases, 1,191 controls), AMR (161 cases, 819 controls), EAS (27 cases, 134 controls), EUR (1,679 cases, 8,589 controls), MID (4 cases, 17 controls), SAS (5 cases, 25 controls). Each panel displays pairwise comparisons of the first three principal components: PC1 vs PC2 (left), PC1 vs PC3 (middle), and PC2 vs PC3 (right).

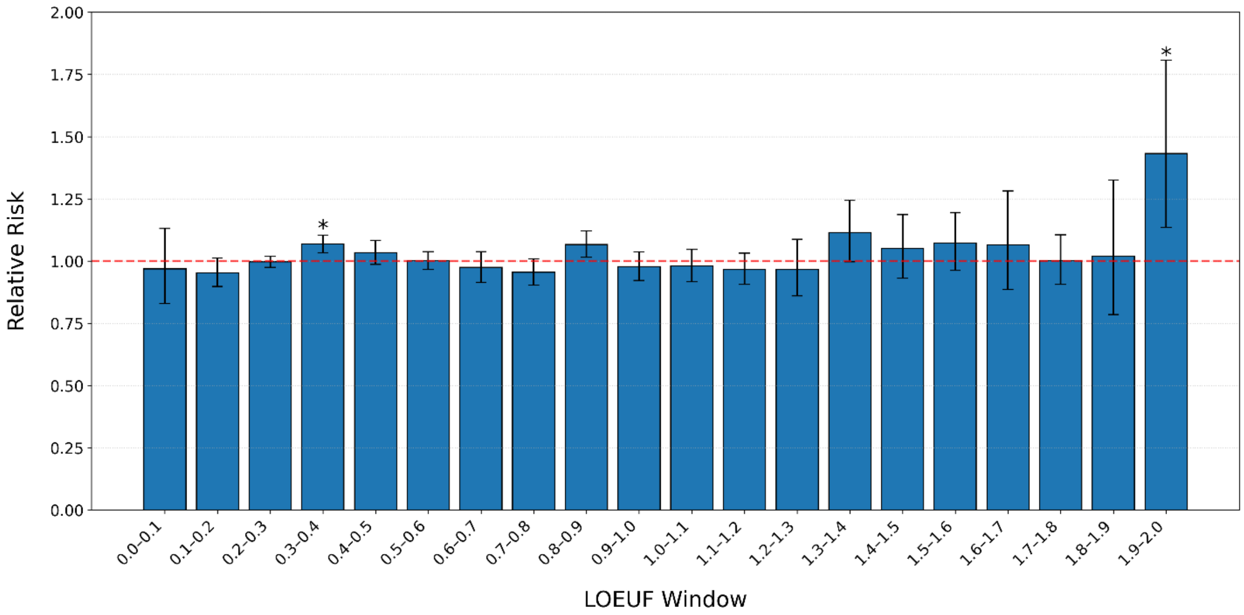

Supplementary Figure 2. Enrichment of rare conserved variants in antisense lncRNA overlap regions across LOEUF quantiles in OCD (European ancestry). Relative risk of rare conserved variants (MAF < 0.1%) in regions where antisense lncRNAs overlap with their sense protein-coding genes, stratified by 20 quantiles of LOEUF scores. Genes are ranked from most constrained (quantile 1, lowest LOEUF) to least constrained (quantile 20, highest LOEUF). Analysis restricted to individuals of European ancestry includes 1,947 OCD cases and 9,937 matched controls. Error bars represent 95% confidence intervals. The dashed horizontal line indicates relative risk = 1 (no effect). Asterisk denotes P < 0.05 by binomial test comparing variant burden between cases and controls.

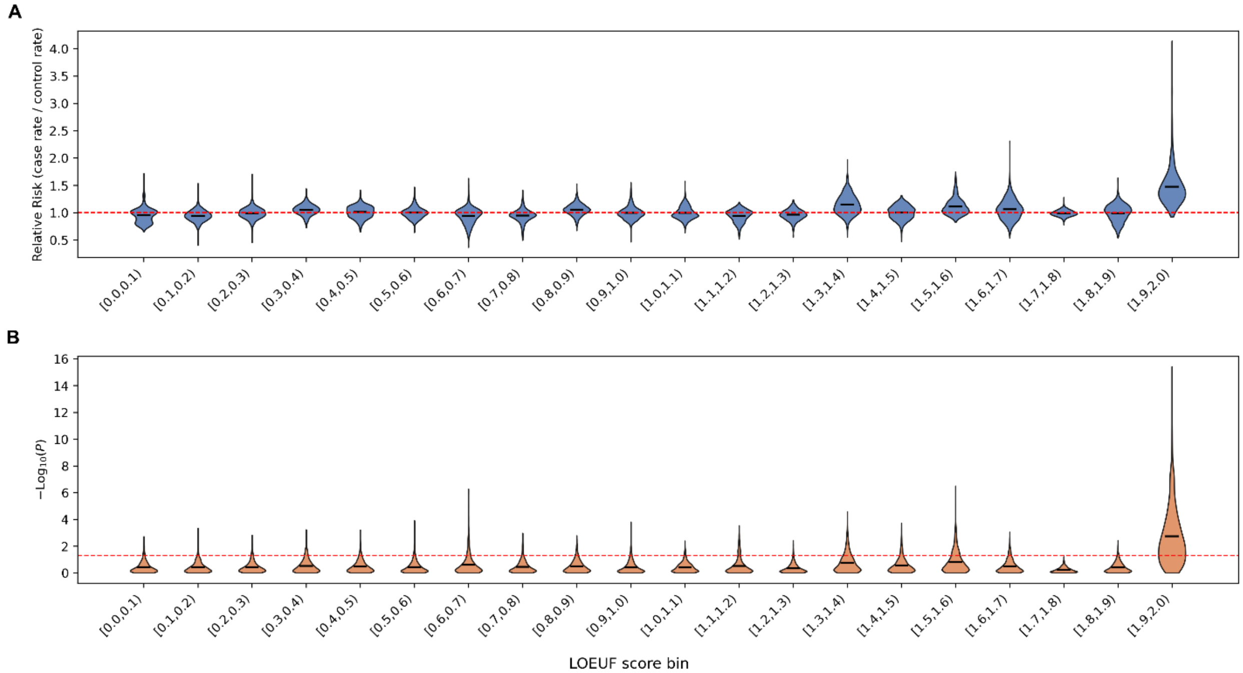

Supplementary Figure 3. Bootstrap analysis equalizing statistical opportunity across LOEUF bins. (A) Relative risk (RR) of rare conserved overlap variants by LOEUF score bin, shown as violin plots summarizing a fixed-K bootstrap with replacement performed separately within each bin (K = 10 genes per iteration). In each bootstrap iteration, K genes were sampled with replacement from the genes present in that LOEUF window, variant counts were aggregated across the sampled genes, and RR was computed as the case carrier rate divided by the control carrier rate (continuity correction applied when needed). The dashed red line marks RR = 1 (no enrichment). (B) Corresponding violin plots of statistical significance reported as $-\log_{10}$(*P* values) from a binomial test comparing the case fraction within the sampled set to the global case fraction. The dashed red line marks *P*=0.05. For both panels, each violin reflects the bootstrap distribution for that LOEUF bin; wider shapes indicate greater dispersion, and the central marker denotes the mean of the bootstrap distribution. X-axis labels indicate LOEUF score bins.

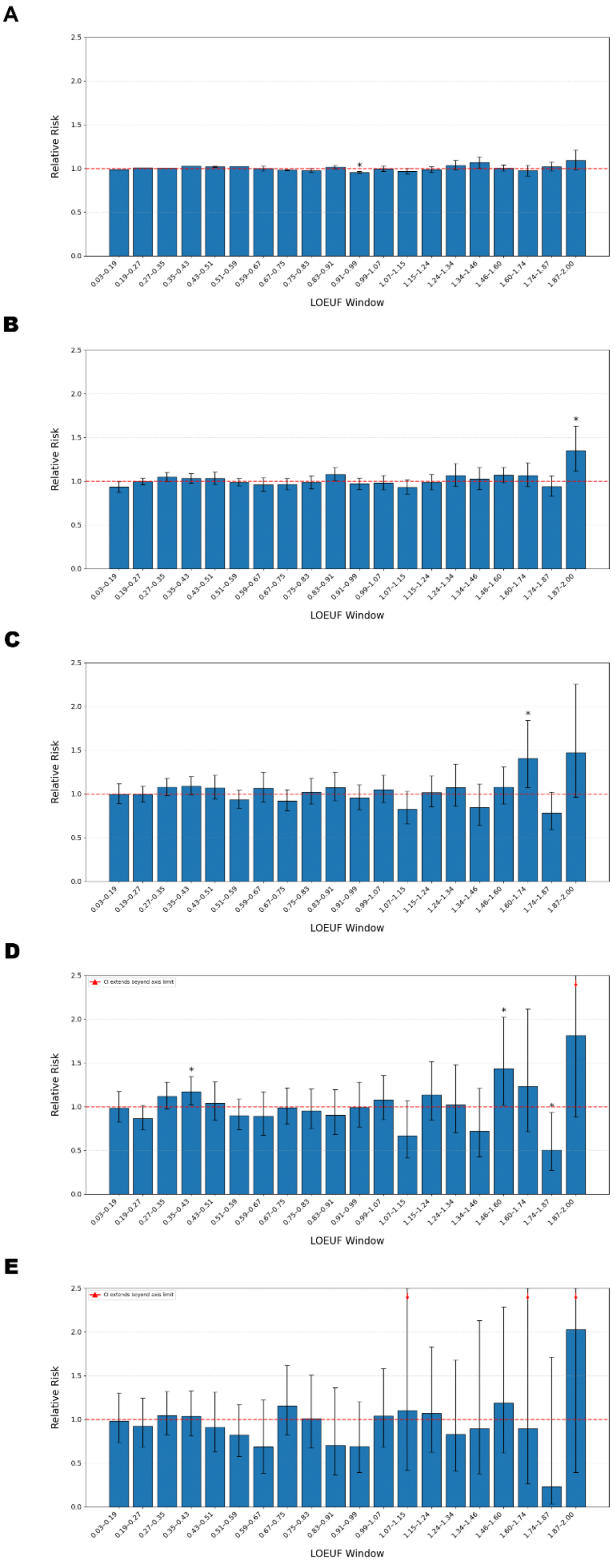
A
Relative Risk
0.0
0.5
1.0
1.5
2.0
2.5
0.03–0.19
0.19–0.27
0.27–0.35
0.35–0.43
0.43–0.51
0.51–0.59
0.59–0.67
0.67–0.75
0.75–0.83
0.83–0.91
0.91–0.99
0.99–1.07
1.07–1.15
1.15–1.24
1.24–1.34
1.34–1.46
1.46–1.60
1.60–1.74
1.74–1.87
1.87–2.00
LOEUF Window
B
Relative Risk
LOEUF Window
C
Relative Risk
LOEUF Window
D
CI extends beyond axis limit
Relative Risk
LOEUF Window
E
CI extends beyond axis limit
Relative Risk
LOEUF Window

Supplementary Figure 4. Rare variant burden in antisense lncRNA overlap regions stratified by gene constraint levels and conservation scores. Analysis of rare variant burden in antisense lncRNA overlap regions across genes stratified by constraint levels. Genes were divided into 20 quantiles based on their LOEUF (Loss-of-function Observed/Expected Upper bound Fraction) scores, where lower LOEUF values indicate greater constraint against loss-of-function variation. (A) Relative risk of rare variants in antisense lncRNA overlap regions across all LOEUF quantiles, showing no conservation filter applied (all variants included regardless of GERP++ score). (B) Relative risk analysis with conservation filter requiring GERP++ score > 0. (C) Relative risk analysis with conservation filter requiring GERP++ score > 1. (D) Relative risk analysis with conservation filter requiring GERP++ score > 2, showing emerging enrichment in the least constrained genes (20th quantile). (E) Relative risk analysis with the most stringent conservation filter (GERP++ score > 3).

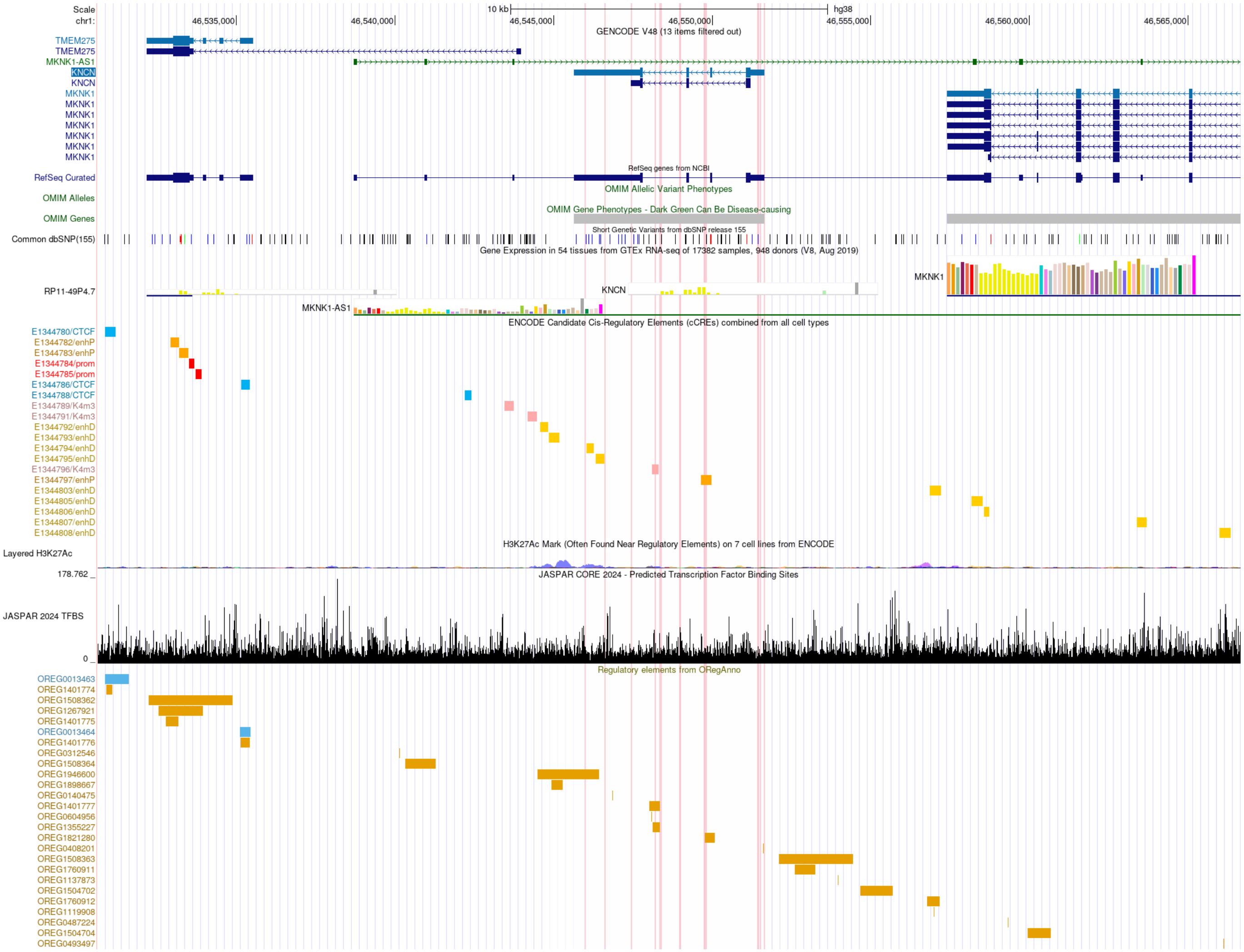

Supplementary Figure 5. Integrated genomic landscape of the *KNCN/MKNK1-AS1* locus revealing regulatory architecture and transcription factor binding sites. Multi-track visualization displaying the genomic region chr1:46,530,623-46,566,664 (hg38/GRCh38). GENECODE v48 and RefSeq gene annotations show *KNCN*, *MKNK1-AS1*, *TMEM275* and *MKNK1* gene structures with exons (thick bars) and introns (thin lines). Common genetic variants from dbSNP build 155 are displayed along with additional transcript annotations. ENCODE candidate cis-regulatory elements (cCREs) are integrated from multiple cell types, with each colored block representing a distinct regulatory element class. H3K27ac ChIP-seq signal (yellow/orange) from seven ENCODE cell lines indicates active enhancer regions. JASPAR 2024 predicted transcription factor binding sites are shown with binding affinity scores (black histogram) and individual TF motifs color-coded by factor identity. Vertical pink lines denote regions including rare conserved variants identified in this study.

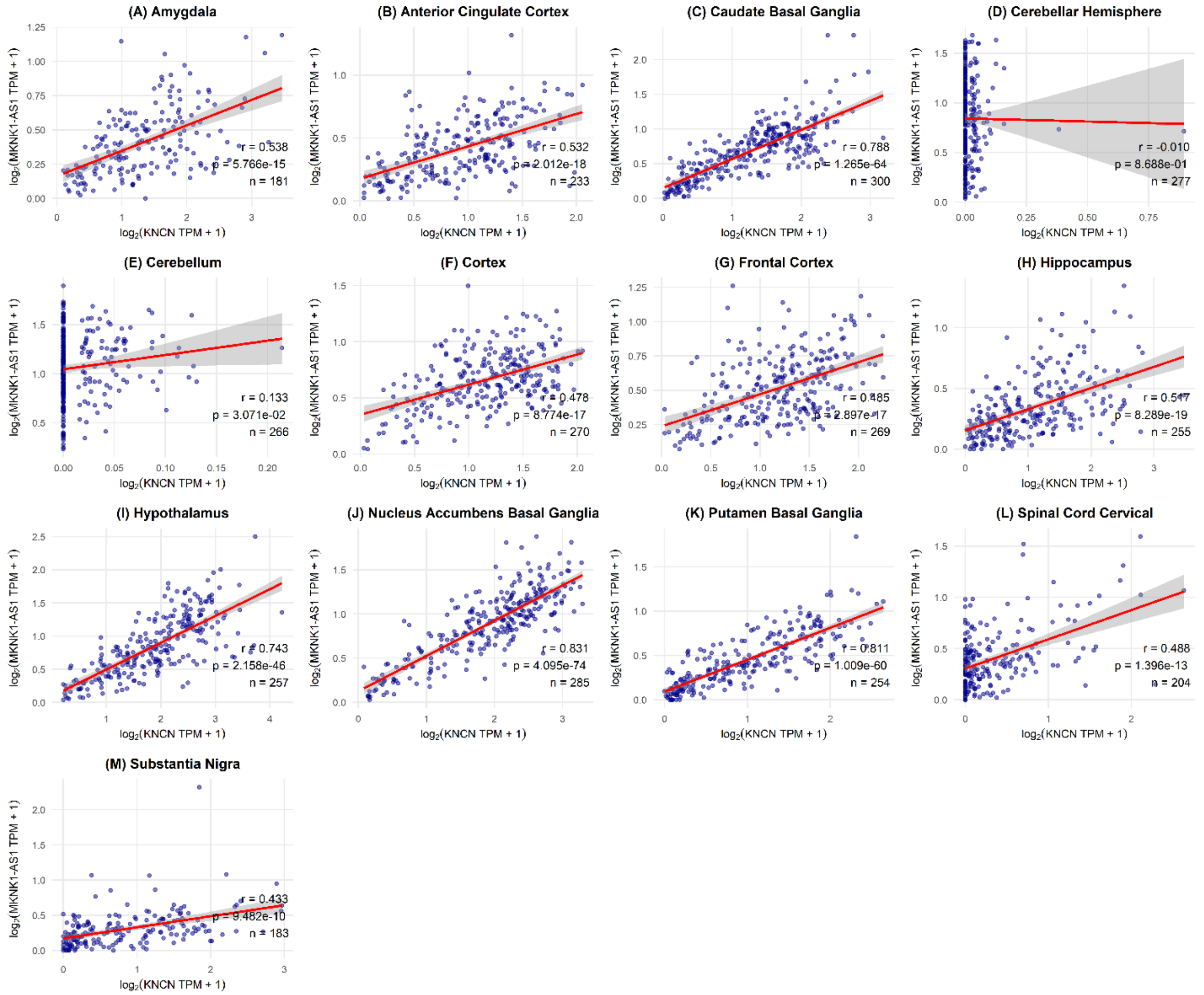

Supplementary Figure 6. Correlation between KNCN and MKNK1-AS1 expression across 13 human brain regions. Scatter plots showing the relationship between log2-transformed expression values (TPM + 1) of KNCN (x-axis) and MKNK1-AS1 (y-axis) in (A) amygdala, (B) anterior cingulate cortex, (C) caudate basal ganglia, (D) cerebellar hemisphere, (E) cerebellum, (F) cortex, (G) frontal cortex, (H) hippocampus, (I) hypothalamus, (J) nucleus accumbens basal ganglia, (K) putamen basal ganglia, (l) spinal cord cervical c-1, and (M) substantia nigra. Each point represents an individual sample from the GTEx v10 database. Red lines indicate linear regression fits with 95% confidence intervals shown in gray shading. Pearson correlation coefficients (r) and p-values are displayed for each brain region. Panels are arranged alphabetically by brain region name.

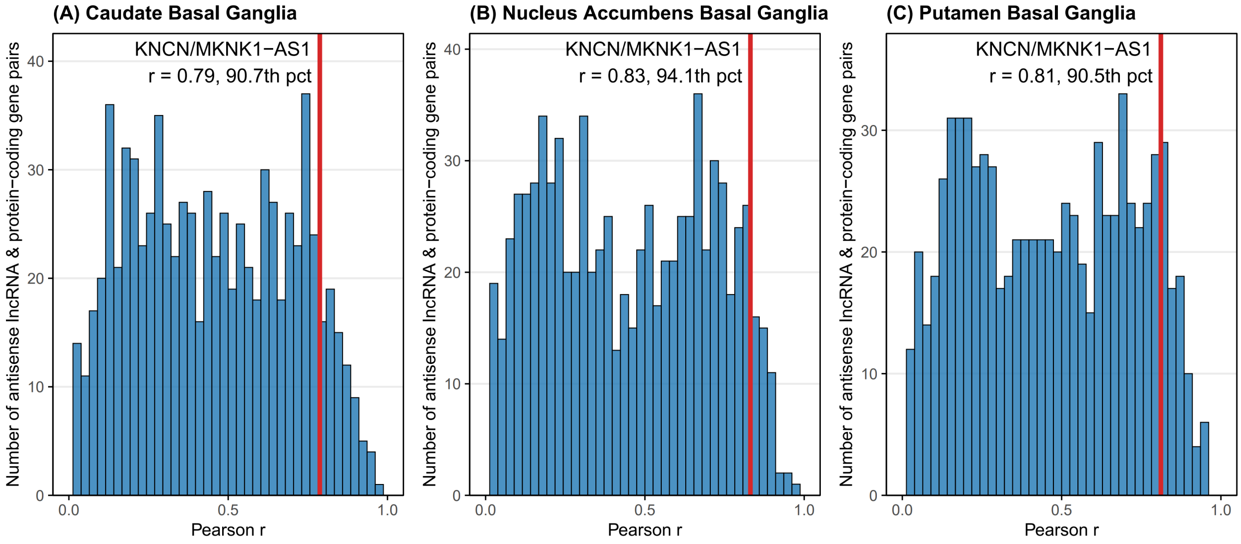

Supplementary Figure 7. Benchmarking striatal co-expression of *KNCN* and *MKNK1-AS1* against other antisense lncRNA–protein-coding gene pairs. Histograms show the distribution of Pearson correlation coefficients (r) between expression of 992 antisense lncRNA–protein-coding gene pairs in three GTEx striatal tissues: (A) caudate nucleus, (B) nucleus accumbens, and (C) putamen. For each tissue, expression of each antisense lncRNA and its paired protein-coding gene was quantified as $\log_2$(TPM + 1) across GTEx donors, and Pearson's r was calculated per pair. Bars indicate the number of antisense lncRNA–protein-coding gene pairs falling into each correlation bin. The red vertical line marks the correlation for the pair *KNCN*/*MKNK1-AS1* in each tissue, and the inset text reports its r value and percentile rank among all 992 pairs (90.7th percentile in caudate, 94.1th percentile in nucleus accumbens, and 90.5th percentile in putamen).

Reference

1. All of Us Research Program Investigators, Denny JC, Rutter JL, Goldstein DB, Philippakis A, Smoller JW, *et al.* (2019): The “All of Us” Research Program. *N Engl J Med* 381: 668–676.
2. Karczewski KJ, Francioli LC, Tiao G, Cummings BB, Alföldi J, Wang Q, *et al.* (2020): The mutational constraint spectrum quantified from variation in 141,456 humans. *Nature* 581: 434–443.
3. Manichaikul A, Mychaleckyj JC, Rich SS, Daly K, Sale M, Chen W-M (2010): Robust relationship inference in genome-wide association studies. *Bioinformatics* 26: 2867–2873.
4. Davydov EV, Goode DL, Sirota M, Cooper GM, Sidow A, Batzoglou S (2010): Identifying a high fraction of the human genome to be under selective constraint using GERP++. *PLoS Comput Biol* 6: e1001025.
5. Lee S, Emond MJ, Bamshad MJ, Barnes KC, Rieder MJ, Nickerson DA, *et al.* (2012): Optimal unified approach for rare-variant association testing with application to small-sample case-control whole-exome sequencing studies. *Am J Hum Genet* 91: 224–237.
6. GTEx Consortium (2020): The GTEx Consortium atlas of genetic regulatory effects across human tissues. *Science* 369: 1318–1330.
7. Strom NI, Gerring ZF, Galimberti M, Yu D, Halvorsen MW, Abdellaoui A, *et al.* (2025): Genome-wide analyses identify 30 loci associated with obsessive-compulsive disorder. *Nat Genet* 57: 1389–1401.
